# Neurocysticercosis: A neglected but preventable cause of seizure in adults

**DOI:** 10.1002/ccr3.8454

**Published:** 2024-01-23

**Authors:** Prosper Adjei, Vida Obese, Richard Tang, Kingsley Owusu Manu, Yaw Owusu Afriyie Boateng, Eunice Ansomah Donkor

**Affiliations:** ^1^ Department of Internal Medicine Methodist Hospital Wenchi Ghana; ^2^ Directorate of Internal Medicine Komfo Anokye Teaching Hospital Kumasi Ghana

**Keywords:** Cysticercosis, epilepsy, focal seizure, Neurocysticercosis, neuroimaging, *Taenia solium*

## Abstract

Neurocysticercosis is an infection of the central nervous system caused by the larval stage of *Taenia solium*. Although endemic in sub‐Saharan Africa, it is neglected but remains a significant cause of preventable seizure in adults. Its diagnosis is challenging and is frequently missed due to its variable clinical manifestations and lack of diagnostic facilities in most areas of sub‐Saharan Africa. This report discusses two cases of parenchymal neurocysticercosis in Ghanaians who presented to the emergency unit of a District Hospital with adult‐onset seizures. The two cases highlight the need for a high index of suspicion and also underscore the important role of neuroimaging in the evaluation of patients presenting with adult‐onset seizures in neurocysticercosis endemic areas. This is necessary for prompt detection and initiation of appropriate therapy in order to improve prognosis.

## Graphical abstract

Contrast enhanced axial CT scan images of the brain showing cystic lesions with eccentric hyperdense foci (cysts with scolices) in both frontal lobes.

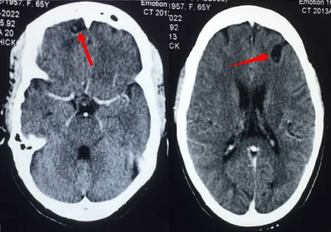

## 1 INTRODUCTION

Neurocysticercosis is an infection of the central nervous system caused by the larval stage of *Taenia solium*.^1^ It mostly affects people in rural communities with poor sanitary conditions and poor pig‐keeping practices.^2^

It is endemic in South and Central America, sub‐Saharan Africa and parts of Asia and accounts for about 30% of epilepsy cases in these areas^2^, ^3^ making it a significant cause of preventable seizure worldwide.^4^ One study found the prevalence of neurocysticercosis among people with epilepsy in sub‐Saharan Africa to be 22%.^5^ In West Africa; however, data on neurocysticercosis are limited with few cases reported in some countries.^6^ For many years, it has been neglected in sub‐Saharan Africa and West Africa because little attention is paid to its surveillance, prevention, and treatment.^6^, ^7^

Human beings and pigs are the hosts involved in the life cycle and transmission of *T. solium*. In most instances, affected individuals ingest *T. solium* eggs shed in the stool of human tapeworm carriers. Autoinfection via fecal‐oral contamination in human tapeworm carriers can also occur. The ingested eggs develop into oncospheres which penetrate the human intestinal wall and circulate through the bloodstream to the central nervous system where they mature into cysticerci, causing neurocysticercosis. Consumption of infected pork leads to taeniasis but not cysticercosis.^8^ The disease evolves through four main phases (Escobar's pathological stages) namely vesicular, colloidal, granular nodular and calcified stages.^9^ It is divided into parenchymal and extraparenchymal forms depending on the anatomic location of the cysticerci within the central nervous system. In extraparenchymal neurocysticercosis, cysticerci can lodge in the ventricles, the subarachnoid space, the spinal cord, and the eyes.^10^

In sub‐Saharan Africa, the diagnosis of neurocysticercosis is fraught with challenges and is often missed due to its variable and nonspecific clinical features as well as lack of diagnostic facilities.^2^, ^7^ A high index of suspicion is therefore required for prompt diagnosis and initiation of treatment in order to improve outcomes.

Given that neurocysticercosis is a significant cause of epilepsy in endemic areas such as sub‐Saharan Africa, the importance of strategies aimed at breaking the life cycle and transmission of *T. solium* cannot be overstated. Good personal hygiene, treatment of human tapeworm carriers, consumption of well cooked pork, improved environmental sanitation, proper disposal of human feces, improved pig husbandry practices as well as vaccination, and anti‐parasitic treatment of pigs are measures that should be implemented to prevent new cases of neurocysticercosis.^2^

In this report, we discuss the cases of two Ghanaians who presented at Methodist Hospital, Wenchi, Ghana with adult‐onset seizures. Both of them were diagnosed with parenchymal neurocysticercosis and treated accordingly.

## 2 CASE PRESENTATION

### 2.1 CASE 1

A 65 year old woman with no significant past medical history was brought to the emergency unit of a District Hospital in Ghana with an episode of seizure. Her daughters had noted repetitive nodding of the head and shaking movements of her left upper limb. This lasted about 1–2 min and it was the first time she had ever had a seizure. The episode was not preceded by an aura. There was no loss of consciousness, fecal or urinary incontinence, slurred speech, weakness in the limbs, loss of sensation in any part of her body, fever or neck pain. There was a 2 month antecedent history of recurrent mild headache. She had no family history of epilepsy or history of foreign travel. She was a resident of a rural community with poor sanitary conditions in the northern part of Ghana. Pigs roamed freely in her community. She was once into pig rearing and also used to be an avid pork eater.

On physical examination, she was afebrile (36.7°C), anicteric, not pale and not wasted. She had no oral thrush or peripheral lymphadenopathy. Random blood sugar was 8.7 mmol/L. Her pulse and blood pressure were 84 beats per minute and 118/72 mmHg respectively. She was conscious and alert with no signs of meningeal irritation. Neurological assessment was unremarkable.

**FIGURE 1:**
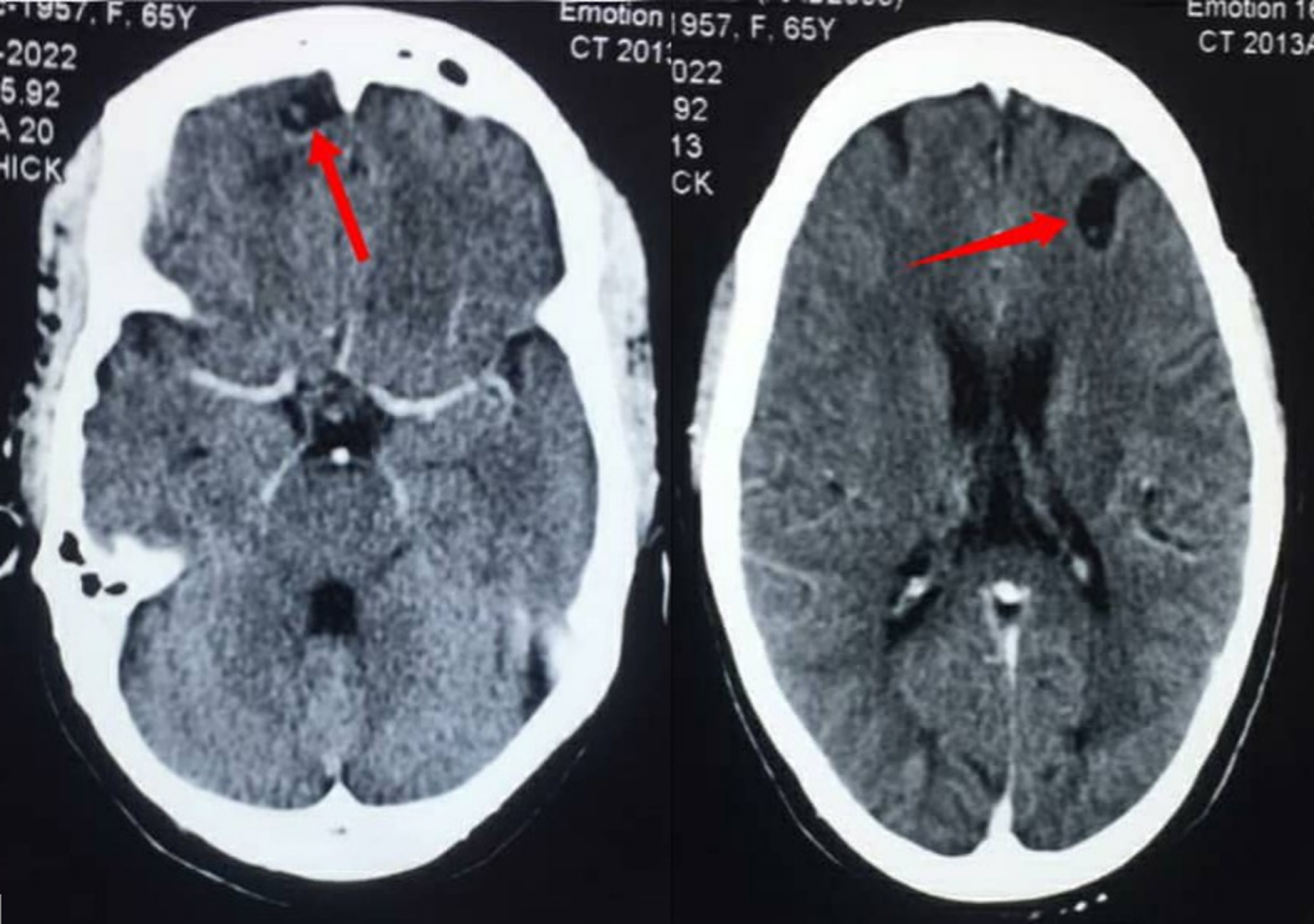
Contrast enhanced axial computed tomography (CT) scan images of the brain showing cystic lesions with eccentric hyperdense foci (cysts with scolices) in both frontal lobes.

**FIGURE 2:**
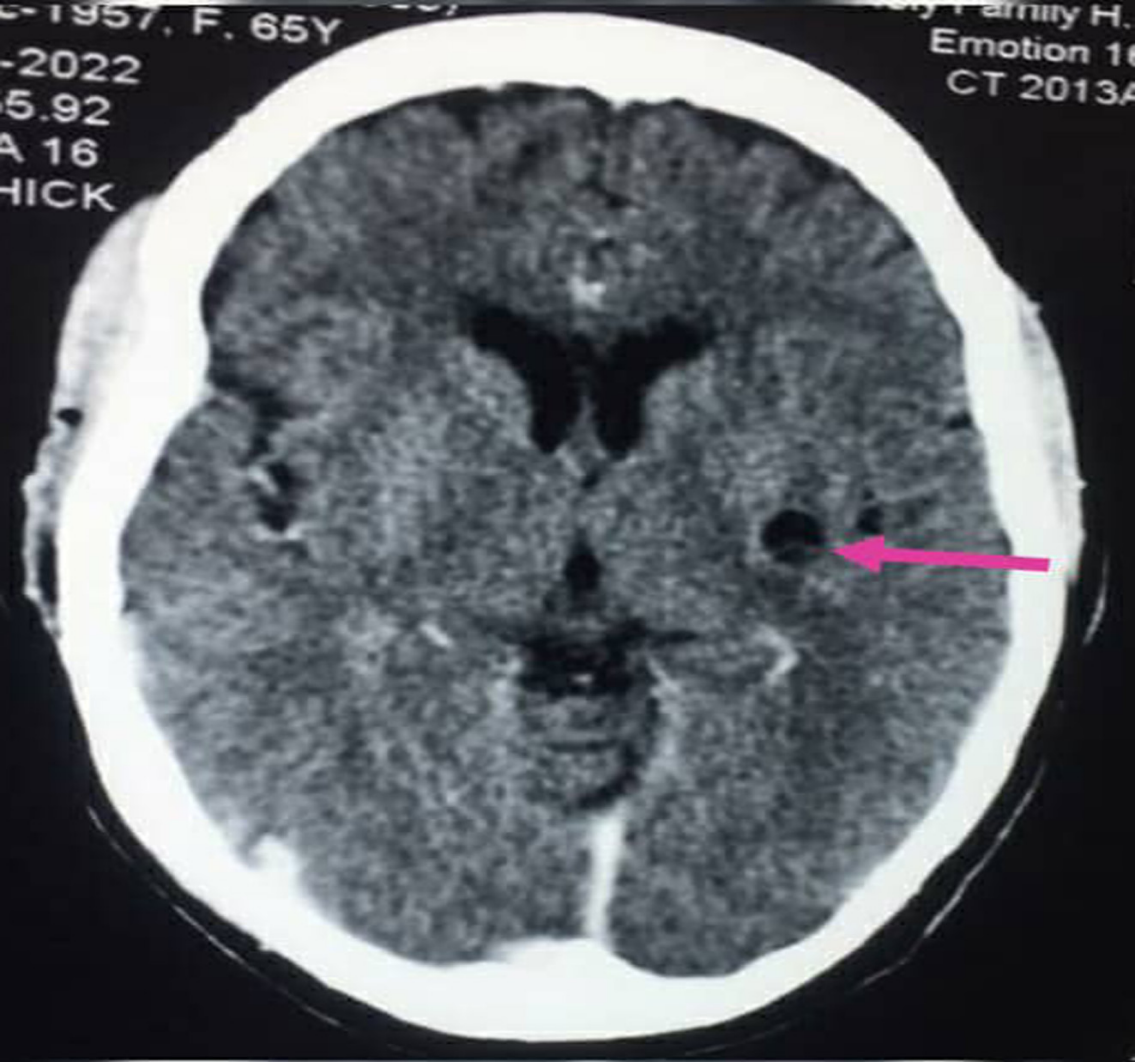
Contrast enhanced axial computed tomography (CT) scan image of the brain showing cyst with scolex in the left insular cortex.

Her full blood count, renal and liver biochemistries as well as serum electrolytes (Ca^2+^, Mg^2+^, Na^+^, K^+^) were normal. She was also HIV negative. Contrast enhanced computed tomography (CT) scan of the brain revealed subcortical cystic lesions without perilesional oedema with eccentric internal hyperdense foci measuring 1.3 × 1.0 cm and 1.8 × 1.2 cm in the right and left frontal lobes respectively (Figure 1). There was a similar lesion in the left insular cortex measuring 1.0 × 0.8 cm (Figure 2).

She was diagnosed with vesicular stage of parenchymal neurocysticercosis. Funduscopic examination was done to exclude papilledema and ocular involvement. She could not afford levetiracetam and was therefore started on carbamazepine. She was also treated with a combination of albendazole and praziquantel for 14 days along with dexamethasone which was started 2 days prior to initiation and continued throughout the duration of anthelmintic therapy. At her last review 24 months after initiation of treatment, she had not had any more seizures.

### 2.2 CASE 2

A 43 year old man with no history of chronic illness was well until the day of presentation when he had an episode of seizure. While the patient was asleep, his relative noted stiffening of his body followed rapidly by rhythmic jerking of all his limbs which lasted about 45 s. He was unresponsive to calls during the episode. There was no fecal or urinary incontinence or foaming at the mouth. He had had two similar episodes of generalized seizures in the preceding 12 months. He was admitted at a clinic when he had the first seizure but was subsequently lost to follow‐up. There was no family history of epilepsy and he had never traveled outside of Ghana. He was a non‐smoker and also drank alcohol occasionally but did not use illicit drugs. He was a regular pork eater and a previous pig farmer. He lived in a community with free‐ranging pigs and limited access to toilet facilities. On arrival at the emergency unit of our hospital, he had another episode of generalized seizure which was aborted with intravenous diazepam.

On physical examination, he was afebrile (37.1°C), anicteric, not pale, not dyspnoeic, and not wasted. There was no tongue bite, oral thrush or peripheral lymphadenopathy. Random blood sugar was 6.6 mmol/L. He had a regular pulse of 63 beats per minute and a blood pressure of 117/63 mmHg. He was fully conscious with no obvious neurologic deficit. His neck was supple with negative Kernig's sign.

**FIGURE 3:**
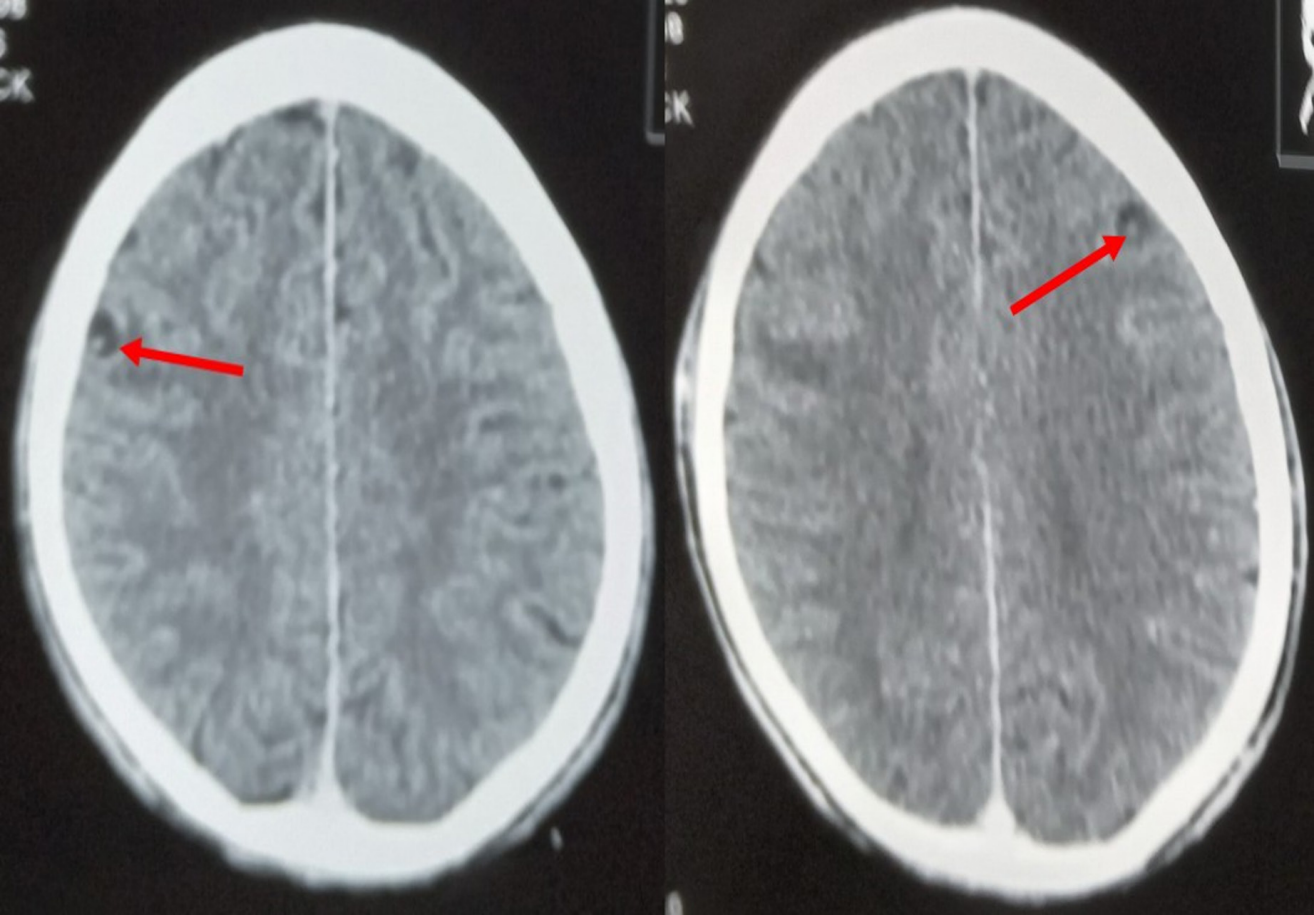
Contrast enhanced axial computed tomography (CT) scan images of the brain showing cysts with eccentric scolices in the frontal lobes.

**FIGURE 4:**
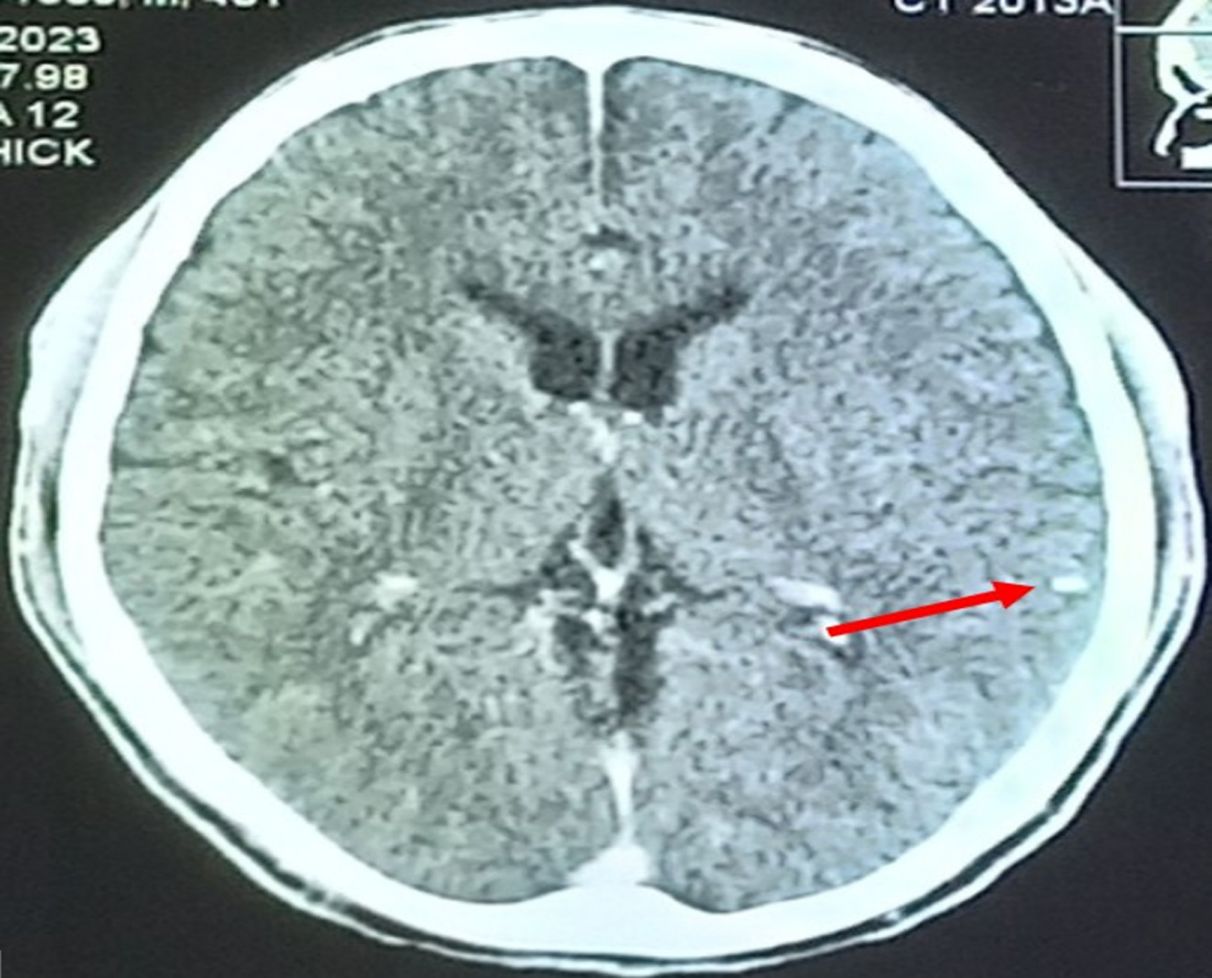
Contrast enhanced axial computed tomography (CT) scan image of the brain showing a calcified nodule in the left parietal region.

Laboratory investigations revealed normal complete blood count, renal and liver biochemistries, serum electrolytes (Ca^2+^, Mg^2+^, Na^+^, K^+^) and negative HIV status. Contrast enhanced CT scan of the head showed rim‐enhancing, cystic lesions with eccentric internal hyperdense foci in both frontal lobes (Figure 3). There was no perilesional edema. Also noted was a calcified cortical nodule in the left parietal region (Figure 4).

A diagnosis of vesicular and calcified stages of parenchymal neurocysticercosis was made. Ocular cysticercosis and papilledema were ruled out with funduscopic assessment. Anti‐seizure treatment with levetiracetam was initiated. He was also started on a 2 week course of albendazole together with dexamethasone which was started 24 h before initiation of albendazole therapy. At follow‐up 2 months after discharge, there had been no recurrence of seizures.

## 3 DISCUSSION

A few cases of neurocysticercosis have been reported in some West African countries like Cote d'Ivoire, Mali and Cape Verde.^6^ To the best of our knowledge, these are the first published case reports of neurocysticercosis in Ghana.

Both patients had never traveled to any other neurocysticercosis endemic country apart from being resident in Ghana. They lived in rural communities with poor sanitary conditions and free‐ranging pigs. Both were also previous pig farmers who consumed pork regularly. These factors predisposed them to developing this parasitic infection. A survey carried out at an abattoir by Addy et al to screen for porcine cysticercosis showed a prevalence rate of 9.73% among pigs drawn from all five regions in the northern part of Ghana^11^ where the first patient lived.

The clinical manifestations of neurocysticercosis vary depending on the number, location and stage of cysticerci as well as the host immune response.^10^ Both patients had vesicular stage of parenchymal neurocysticercosis. In addition, the second patient had a calcified nodule in the left parietal region. Although the vesicular stage is usually asymptomatic due to the minimal inflammatory changes produced, our patients had symptoms. Most patients with parenchymal neurocysticercosis develop focal seizures^12^ as observed in our first patient. Generalized seizures may also occur^12^ as seen in our second case. Other clinical features include headache, altered sensorium and hydrocephalus.^2^, ^10^

The diagnosis of neurocysticercosis is based on clinical features, epidemiologic exposure and neuroimaging findings.^2^, ^10^ Magnetic resonance imaging is the modality of choice. CT is the recommended alternative if magnetic resonance imaging is unavailable or contraindicated.^2^ In our case, CT scan of the brain was done because it is comparatively cheaper and readily available in the part of our country where the patients were seen. Although other causes of ring‐enhancing lesions such as tuberculoma, cerebral abscess, cerebral toxoplasmosis, cerebral cryptococcoma and cerebral metastasis were initially considered, the presence of a cystic lesion with eccentric scolex (cyst with dot sign) as occurred in our patients is pathognomonic for the vesicular stage of parenchymal neurocysticercosis.^9^ Other diagnostic tests include histological examination of biopsied brain lesions and enzyme‐linked immunoelectrotransfer blot.^13^ The presence of cyst with scolex on neuroimaging is an absolute criterion for a definitive diagnosis of neurocysticercosis^13^ and obviated the need for brain biopsy in our patients due to the invasive nature of the latter.

Funduscopic examination to exclude ocular cysticercosis is required in all patients prior to the initiation of anthelmintic therapy because inflammation around degenerating cysticerci in the eyes can be sight‐threatening. Acute symptoms like seizures and increased intracranial pressure should be treated first before initiation of anthelmintic therapy. Carbamazepine, phenytoin or levetiracetam should be given even in the case of a single episode of seizure since lesions serve as a focus for recurrent seizures.^2^, ^10^, ^14^ Levetiracetam has fewer side effects and is also better tolerated but the first patient could not afford it.

Anthelmintic therapy is indicated only in patients with viable and/ or degenerating cysts. Patients with more than two cysts are treated with a combination of albendazole (15 mg/kg/day in two divided doses) and praziquantel (50 mg/kg/day in three divided doses) for 10–14 days in most cases of parenchymal neurocysticercosis just as was done for our first patient. Albendazole (15 mg/kg/day in two divided doses) monotherapy is indicated in those with one to two cysts as was the case in the second patient. Adjunctive corticosteroid like dexamethasone or prednisolone should be given prior to initiation and throughout the duration of anthelmintic therapy to reduce seizures caused by anthelmintic therapy‐induced degeneration of viable cysts.^2^, ^10^

Following completion of anthelmintic therapy, repeat neuroimaging is done every 6 months until there is resolution of the cystic lesions. For individuals with multiple lesions, antiseizure therapy is tapered and discontinued after being seizure‐free for 24 consecutive months.^2^, ^4^

## 4 CONCLUSION

Neurocysticercosis is a neglected but significant cause of preventable seizure in sub‐Saharan Africa. A high index of suspicion is required by clinicians for prompt and accurate diagnosis particularly in patients presenting with adult‐onset seizures in endemic areas. Investments in neuroimaging facilities by governments in sub‐Saharan African countries will enhance its early detection and initiation of appropriate treatment in order to improve outcomes.

## AUTHOR CONTRIBUTIONS

**Prosper Adjei:** Conceptualization; investigation; writing – original draft. **Vida Obese:** Writing – review and editing. **Richard Tang:** Data curation. **Kingsley Owusu Manu:** Data curation. **Yaw Owusu Afriyie Boateng:** Data curation. **Eunice Ansomah Donkor:** Data curation.

## FUNDING INFORMATION

The authors received no financial support for the authorship and/or publication of this article.

## CONFLICT OF INTEREST STATEMENT

The authors declare no potential conflicts of interest with respect to the authorship and/or publication of this article.

## ETHICS STATEMENT

Our institution does not require ethical approval for reporting individual cases or case series.

## CONSENT

Written informed consent was obtained from both patients for their anonymized information to be published in this article.

## Data Availability

Data sharing is not applicable.
